# Genetic Analysis of the Plasmid-Based Temperature-Lethal Mutant *pa1792|lpxH(Ts)* in *Pseudomonas aeruginosa*

**DOI:** 10.3390/genes15060784

**Published:** 2024-06-14

**Authors:** Haoyang Zhang, Zhili Yang, Jianhua Liu

**Affiliations:** Systems Biology, School for Marine Science and Technology, Zhejiang Ocean University, Zhoushan 316022, China; zhanghaoyang@zjou.edu.cn (H.Z.); yangzhili@zjou.edu.cn (Z.Y.)

**Keywords:** conditional allele, essential gene, lipid A, LpxH, *Pseudomonas aeruginosa*

## Abstract

Many enzymes in the Raetz pathway for lipid A biosynthesis in *Escherichia coli* are essential. A homologous protein Pa1792|LpxH in *Pseudomonas aeruginosa* is known to complement the loss of LpxH in *E. coli*. Genome-wide transposon-insertion sequencing analysis indicates that *lpxH* is essential in *P. aeruginosa*. However, genetic analysis of *lpxH* in *P. aeruginosa* has not been carried out, partly because the conditional alleles of essential genes are not readily constructed. In this study, we first constructed a plasmid-based temperature-sensitive mutant *ΔlpxH/pTS-lpxH* or *lpxH(Ts)* in *P. aeruginosa* PAO1. Spot-plating assay indicated that *lpxH(Ts)* was lethal at a restrictive temperature, confirming its essentiality for growth. Microscopic analysis revealed that *lpxH(Ts)* exhibited an oval-shaped morphology, suggesting that *lpxH* was required for rod-shape formation. SDS-PAGE and Western blotting analysis showed that *lpxH(Ts)* failed to synthesize lipid A, consistent with its function in lipid A biosynthesis. Strong expression of *lpxH* but not the non-homologous isoenzyme *lpxI* or *lpxG* impeded growth and caused cell lysis, implying that *lpxH*-specific cofactors were required for this toxic effect in *P. aeruginosa*. Together, our results demonstrate that *lpxH* is essential for lipid A biosynthesis, rod-shaped growth, and viability in *P. aeruginosa*. We propose that this plasmid-based conditional allele is a useful tool for the genetic study of essential genes in *P. aeruginosa*.

## 1. Introduction

*Pseudomonas aeruginosa* is a rod-shaped Gram-negative bacterium that is widely found in soil and water. It is an opportunistic pathogen that attacks immunocompromised patients. Due to its intrinsic antibiotic resistance, it has been a top killer in intensive care units [1]. The World Health Organization has listed it as one of the three top pathogens critically required for research and the development of new antibiotics [2].

Lipopolysaccharide (LPS), also known as endotoxin, is a major non-proteinaceous component of the cell wall in most of the Gram-negative bacteria [3,4,5,6]. LPS consists of three domains: lipid A, core oligosaccharide, and O-specific antigen polysaccharide or O-antigen, in which lipid A serves as an anchor on the outer leaflet of the outer membrane in the Gram-negative bacterial cell envelope [3,4,5,6].

The lipid A biosynthesis pathway also known as the Raetz pathway consists of nine enzymes involved in the biosynthesis of Kdo_2_-lipid A in *Escherichia coli* [3,7]. All of the first six enzymes, namely LpxA, LpxC, LpxD, LpxH, LpxB, and LpxK, are essential for synthesizing the minimal structure lipid IV_A_ needed for viability in many Gram-negative bacteria [3,7,8]. It has been an attractive target for the development of antibiotics [9,10].

Many Gram-negative bacteria contain homologous sequences with LpxA, LpxC, LpxD, LpxB, and LpxK in *E. coli*, but not LpxH [11]. It has been shown that some bacteria contain non-homologous isofunctional sequences of LpxI or LpxG, instead of LpxH [11,12]. Genetic analysis indicates that the sequence of *lpxI* from *Caulobacter crescentus* [11] or *lpxG* from *Chlamydia trachomatis* is capable of complementing the loss of *lpxH* function in *E. coli* [12]. However, genetic analysis of *lpxH* mutants in *P. aeruginosa* has not been performed, largely caused by conditional alleles that may not be readily obtained. To circumvent this issue, we have adopted the three-step protocol for the construction of the plasmid-based conditional lethal allele of essential genes in *P. aeruginosa* [13,14].

In this study, we show that the plasmid-based temperature-sensitive mutant *ΔlpxH/pTS-lpxH* or *lpxH(Ts)* that we constructed was lethal at the restrictive temperature, confirming its essentiality for growth. Genetic phenotypes of the *lpxH(Ts)* mutant are presented and their implications are discussed. Together, our results demonstrate that the plasmid-based conditional lethal allele is a useful tool for the genetic analysis of essential genes in *P. aeruginosa*.

## 2. Materials and Methods

### 2.1. DNA, Plasmids, and Bacterial Cultures

The oligonucleotides, plasmids, and bacterial strains used in this study are shown in Table 1. Strains are cultivated in LB, 1 L of which contains: 10 g tryptone (Cat# LP0042B, Oxoid, Hampshire, UK), 10 g NaCl, 5 g yeast extract (Cat# LP0021B, Oxoid), pH 7.0 liquid or solid medium supplemented with antibiotics (100 μg mL^−1^ ampicillin, 50 μg mL^−1^ gentamicin, and 100 μg mL^−1^ tetracycline), and chemicals such as 10% sucrose (Cat# H-10021463, Sinopharm, Beijing, China) or 0.2% arabinose (Cat# A106195, Sinopharm) at 30 °C or 42 °C as indicated.

### 2.2. Plasmid Construction

We used the same deletion plasmid and rescue plasmid (or TS plasmid) constructed in the previous study [13,14]. For the construction of the *lpxH* deletion and rescue plasmids, the deletion cassette and rescue cassette of *lpxH* were cloned in a deletion plasmid and a rescue plasmid using the homologous recombinase cloning kit (ClonExpress II one-step cloning kit; Vazyme, Nanjing, China). For the construction of overexpression plasmids, the araC-P_BAD_ promoter fragment and downstream gene fragment were cloned in the pBBR1MCS-5 plasmid [15] using the Vazyme cloning kit.

### 2.3. Plasmid-Based ts-Mutant Strain Construction

We used a three-step protocol that we developed previously [13,14] to construct the plasmid-based *ts*-lethal mutant strain *Δpa1792/pTS-pa1792*.

### 2.4. Spot-Plating Assay

The spot-plating assay [16] was adopted to test sensitivities to temperature. In brief, 10-fold serial-diluted cultures were transferred using a 48-pin replicator (V&P Scientific, Inc., San Diego, CA, USA) onto LB plates supplemented with appropriate stress factors and incubated at 30 or 42 °C as indicated.

### 2.5. Fluorescence Microscopic Analysis

Cell morphology was investigated under the Olympus BX53 microscope (Olympus, Tokyo, Japan) using the phase contrast configuration. Fluorescent dyes DAPI (4′,6-diamidino-2-phenylindole) (Cat# 28718-90-3, Sinopharm) and Nile red (7385-67-3, Sinopharm) were used to visualize the DNA and cytoplasmic membrane, respectively.

### 2.6. LPS Preparation

A standard method of LPS preparation by Hitchcock and Brown [17] was followed. Briefly, 1 mL of fresh culture (0.8 OD_600_) was harvested and pelleted. The cell pellets were resuspended in 250 μL lysis buffer (1.0 M Tris, pH 6.8, 10% vol/vol glycerol, 2% wt/vol SDS, 4% β-mercaptoethanol) and boiled at 96 °C for 10 min. Proteinase K (Cat# 39450-01-6, Sinopharm) was added to each sample to a final concentration of 200 mg/mL prior to incubation at 55 °C for 4 h. Subsequently, the sample temperature was raised to 70 °C for 1 h to inactivate proteinase K and prepare it for SDS-PAGE analysis.

### 2.7. SDS-PAGE and Silver Staining

LPS samples were resolved on the discontinuous (12% and 15%) gradient SDS-PAGE gel (1.0 mm thickness, 15 wells) to detect O-antigen, core oligosaccharides, and lipid A. Gels were stained by the silver staining method of Fomsgaard et al. [18] before being imaged on a Gel Doc system (Bio-Rad, Hercules, CA, USA).

### 2.8. Western Blotting Analysis

LPS samples in PAGE gels were transferred to PVDF (polyvinylidene difluoride) membranes (0.22-μm pore size, Millipore, Billerica, MA, USA) by means of wet transfer electroblotting at 230 mA for 1 h. The top portion of the membrane corresponding to the 15% SDS-PAGE gel was blotted with monoclonal antibodies (MAb) MF15-4 against O-antigens (Cat# MM-76605-100, MediMabs, Montréal, QC, Canada). The bottom portion corresponding to the 15% SDS-PAGE gel was blotted with mAb 26-5 against lipid A (Cat# ab8467, Abcam, Cambridge, UK) and re-blotted with mAb 5C7-4 against the inner core (Cat# MM-0262-P, MediMabs, Canada) after stripping. Rabbit anti-mouse horseradish peroxidase (HRP)-conjugated secondary antibodies (K1031R-HRP, Solarbio, Beijing, China) were used to visualize the O-antigen, lipid A, and inner core by using an ECL reagent (Millipore) and Tanon 5200 image analyzer (Tanon, Shanghai, China).

### 2.9. Spontaneous Mutagenesis Screening

Approximately 1.0 × 10^+09^ *Δpa1792/pTS-pa1792* cells (i.e., colony forming units calculated using a method reported by Zhang et al. [19]) were plated via spreading onto LB plates and incubated at the semi-restrictive temperature of 40 °C for 2 weeks for suppressor cells through spontaneous mutations as previously described [13,14,20]. Suppressor colonies were validated via streaking on fresh LB plates and incubation at 42 °C. PCR assays using sequence-specific primers for *pa1972* alleles were performed for the validation of the *pa1792* deletion allele.

## 3. Results

### 3.1. Plasmid-Based Temperature-Sensitive Mutant lpxH(Ts) Is Lethal at the Restrictive Temperature in P. aeruginosa

**Figure 1:**
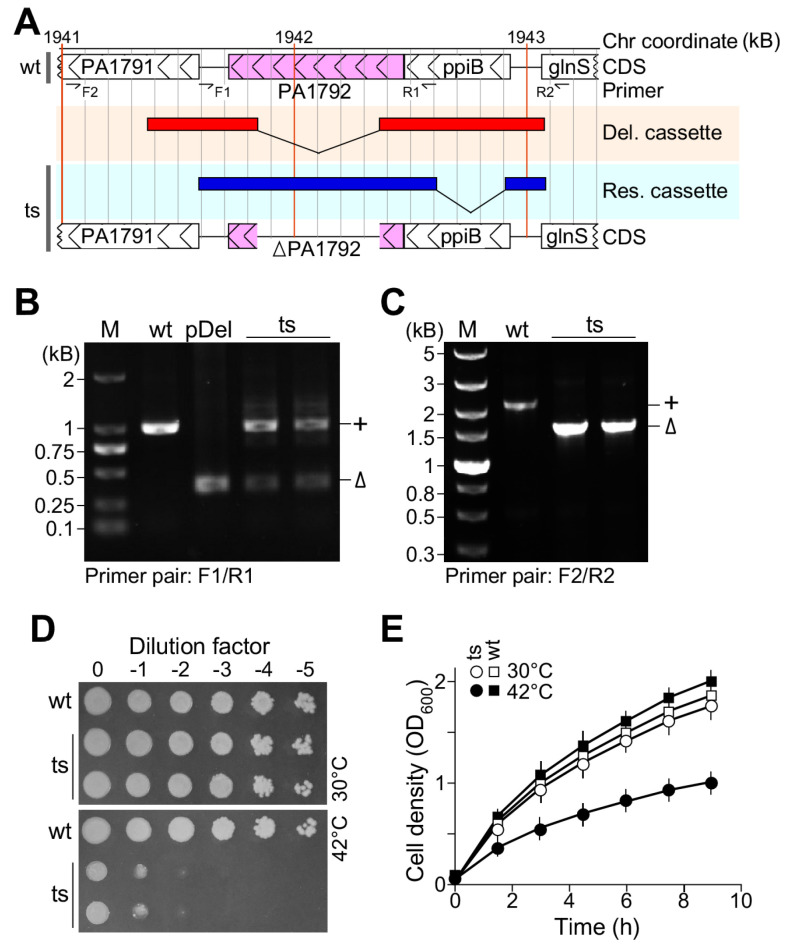
Analysis of plasmid-based *ts*-mutant *lpxH(Ts)* reveals its essentiality for growth. (**A**) A schematic map of the *pa1797|lpxH* locus in *P. aeruginosa* PAO1. Locations of CDS, primer, deletion allele, native promoter controlled wild type allele are shown. (**B**) PCR assay using primer pair F1/R1 specific to *lpxH* alleles in both chromosome and plasmid. (**C**) PCR assay using primer pair F2/R2 specific to *lpxH* alleles in chromosome only. (**D**) Spot-plating assay showing *lpxH(Ts)* growth defect at 42 °C. (**E**) Growth curve analysis showing *lpxH(Ts)* growth defect at 42 °C.

To investigate the genetic phenotype of the essential gene *pa1792|lpxH* in *P. aeruginosa*, we constructed the plasmid-based temperature-sensitive mutant by using a three-step protocol [13,14]. In brief, a schematic map of the deletion plasmid pUC-sacB-gen^R^ containing the *lpxH* deletion allele and permissive temperature rescue plasmid pTS-oriTS-tet*^R^* containing the native promoter controlled wild type allele of *lpxH* were shown in Figure 1A. PCR analysis confirmed that the deletion plasmid contained no *lpxH* wild type allele (Figure 1B, see lane pDel) and both the deletion allele *ΔlpxH* and wild type allele *lpxH^+^* were present in the *lpxH(Ts)* mutant (Figure 1B, see lanes ts) when primer pair F1/R1 was applied. On the other hand, the analysis confirmed that the deletion allele *ΔlpxH* was only present on the chromosome (Figure 1C, see lanes ts) when the primer pair F2/R2 was used. The spot-plating assay with serial-diluted cells indicated that the ts-mutant *lpxH(Ts)* but not the wild type failed to grow at the restrictive temperature of 42 °C (Figure 1D), confirming its essentiality for growth [21]. The growth curve analysis was consistent with the spot-plating assay (Figure 1E), concluding that the plasmid-based temperature-sensitive allele of the essential genes is suitable for genetic analysis in *P. aeruginosa*.

### 3.2. lpxH(Ts) Exhibits an Oval-Shaped Morphology at the Restrictive Temperature

**Figure 2:**
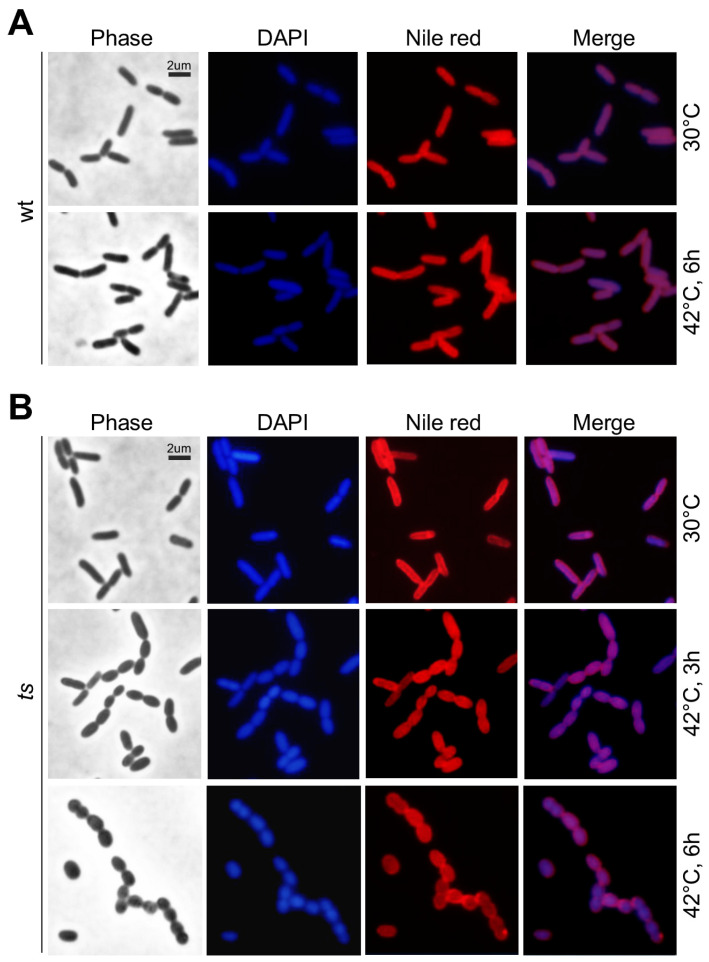
Microscopic analysis showing *lpxH(Ts)* oval-shaped morphology at 42 °C. DAPI and Nile red stain DNA (blue) and plasma membrane (red), respectively. Scale bar of 2 μm is shown. (**A**) Morphology of wild type cells at 30 °C and 42 °C. (**B**) Morphology of *lpxH(Ts)* at 30 °C and 42 °C.

Subsequently, we performed microscopic analysis to assess the terminal morphological phenotype of *lpxH(Ts)* at the restrictive temperature. As a control, we showed that the rod-shaped morphology of wild type cells at 30 °C was hardly altered after growth at 42 °C (Figure 2A). The cellular morphology of the mutant *lpxH(Ts)* cells at the permissive temperature resembled that of the wild type (Figure 2B, see top row). However, 3 h after growth at the restrictive temperature, *lpxH(Ts)* exhibited an oval-shaped morphology (Figure 2B, see mid row). We found that 6 h after growth at the restrictive temperature, some of the *lpxH(Ts)* cells displayed the nearly round-shaped morphology (Figure 2B, see bottom row). This result indicates that *lpxH* is required for rod-shape morphogenesis in *P. aeruginosa*. 

### 3.3. Growth Defect of lpxH(Ts) at the Restrictive Temperature Is Rescued by the araC-P_BAD_ Promoter-Controlled E. coli lpxH or ec.lpxH at the Leaky Expression Level

It was shown that the *P. aeruginosa* LpxH protein or Pa.LpxH shared 47% identity with that of Ec.LpxH [22,23], a UDP-2,3-diacylglucosamine hydrolase involved in the lipid A biosynthetic pathway [22]. Babinski et al. [22] showed that the *pa.lpxH* sequence could compensate for the loss of *E. coli lpxH*. However, it was not known if the *ec.lpxH* sequence could complement the loss of *P. aeruginosa lpxH*. Hence, we investigated if the *ec.lpxH* sequence could compensate for the loss of *P. aeruginosa lpxH*.

**Figure 3:**
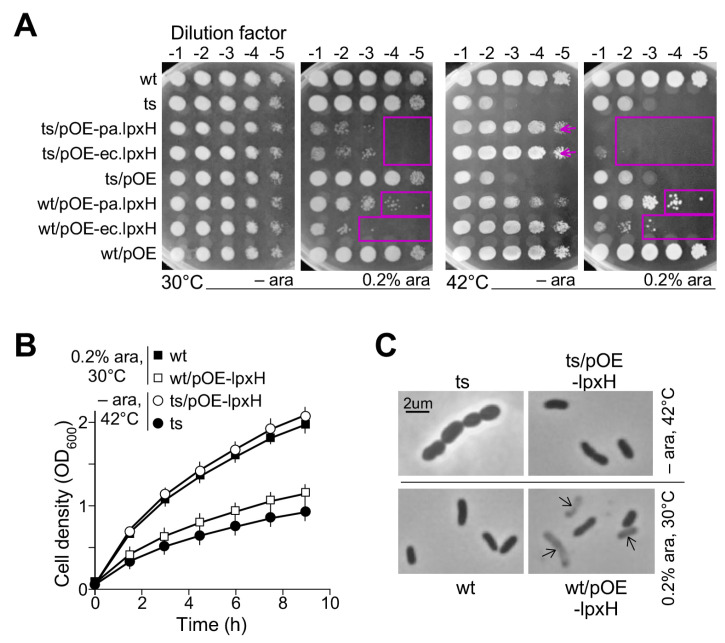
Leaky but not strong expression level of *E. coli lpxH* rescues *lpxH(Ts)* at 42 °C in *P. aeruginosa*. (**A**) Spot-plating assay. Leaky expression level of *lpxH* rescues *lpxH(Ts)* at 42 °C (see arrow). Strong expression level of *lpxH* impedes cell growth (see rectangle). (**B**) Growth curve analysis. X- and Y-axes indicate time (h) and cell density (OD_600_). (**C**) Cell morphology analysis. Arrow shows the lysed cells.

To this end, the *ec.lpxH* coding sequence PCR-amplified from *E. coli* K12 strain was cloned under the control of the araC-P_BAD_ promoter [24] in the multi-host pBBR1MCS-5 plasmid [15] to generate the pOE-ec.lpxH plasmid. The spot-plating assay indicated that under no inducer arabinose, the multi-host plasmid pOE-ec.lpxH at the leakage expression level was sufficient to rescue the growth defect of *lpxh(Ts)* at 42 °C, similar to the positive control plasmid pOE-pa.lpxH (Figure 3A, see arrow). The negative control plasmid pOE showed no effect. This result validated that *P. aeruginosa lpxH* was a functional ortholog of *E. coli lpxH*. On the other hand, we found that strong overexpression (i.e., with 0.2% arabinose supplementation) of *ec.lpxH* or *pa.lpxH* impeded the growth of both the ts-mutant *lpxH(Ts)* and the wild type cells (Figure 3A, see rectangle). 

The growth curve analysis supported the observation that the leaky expression level of *lpxH* rescued the growth of the ts-mutant *lpxH(Ts)* at the restrictive temperature and strong overexpression impeded the growth of the wild type (Figure 3B). Microscopic analysis showed that the oval-shaped morphology of *lpxH(Ts)* at the restrictive temperature was restored to the rod-shaped morphology by the leakage expression level of *ec.lpxH-OE* (Figure 3C, see upper right penal). On the other hand, some rod-shaped cells appeared to be lysed upon the strong expression of *ec.lpxH* (Figure 3C, see bottom right penal). These results indicated that the level of *lpxH* was tightly controlled; without LpxH, it was lethal with oval-shaped terminal morphology and too much LpxH was also lethal, displaying lysed rod-shaped cells.

### 3.4. Lipid A Is Missing in lpxH(Ts) at the Restrictive Temperature in P. aeruginosa

Ec.LpxH, a UDP-2,3-diacylglucosamine hydrolase in *E. coli*, was essential for lipid A biosynthesis [22]. To investigate whether or not lipid A was synthesized in *lpxH(Ts)* at the restrictive temperature in *P. aeruginosa*, we performed SDS-PAGE and Western blotting analysis (see Section 2). For this reason, the wild type and *lpxH(Ts)* mutants cultivated at 30 °C and 42 °C were collected for LPS preparation using the HB method [17]. Based on our previous study [14], while O-antigen signals were found above the position of the 35 kD pre-stained protein marker, core oligosaccharide signals were positioned below the 20 kD protein marker. It should be noted that protein molecular weight markers were not meant for the estimation of the LPS molecular weight but for position alignment of LPS molecules in SDS-PAGE gels and Western blots.

**Figure 4:**
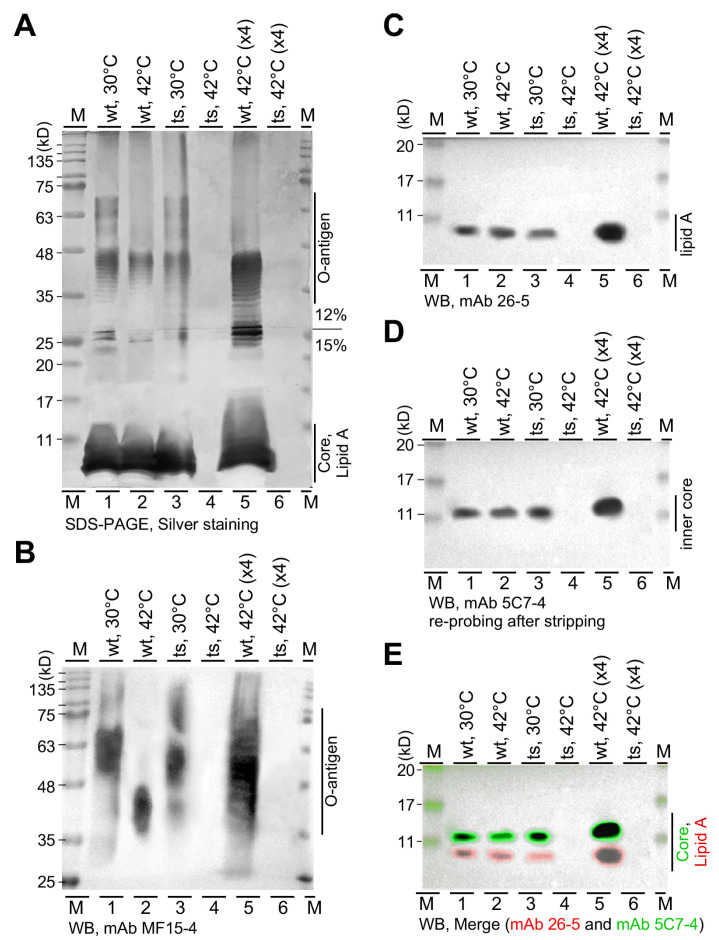
LpxH is required for lipid A biosynthesis in *P. aeruginosa*. (**A**) A silver-stained SDS-PAGE gel image. LPS components including lipid A, core oligosaccharide, and O-antigen are separated on a discontinued gradient SDS-PAGE gel. (**B**) Western blot image. Molecules derived from the upper 12% SDS-PAGE gel portion are probed with mAb MF15-4 against O-antigen. (**C**) Western blot images. Molecules derived from the bottom 15% SDS-PAGE gel portion are probed with mAb 26-5 against lipid A. (**D**) Image of the re-probed blot in (**C**). The blot is re-probed with mAb 5C7-4 against the inner core oligosaccharide after stripping. (**E**) The merge of the blot (**C**) in red and (**D**) in green.

To permit all LPS components such as O-antigen, core oligosaccharide, and lipid A to be displayed in a single gel, we utilized a discontinued (12% and 15%) gradient SDS-PAGE gel, in which the gel electrophoresis was stopped when the pre-stained 25 kD protein marker migrated to the boundary between the 12% and 15% SDS-PAGE gels. Subsequently, the gel was subjected to silver staining after electrophoresis (Figure 4A). We noticed that the LPS sample prepared from the *lpxH(Ts)* mutant at 42 °C hardly showed any silver staining signals. To ensure an unbiased LPS preparation, cell lysates with equal amounts of total proteins were utilized for LPS extraction. To ascertain that the levels of LPS components such as O-antigen, core oligosaccharide, and lipid A were high enough for detection in Western blotting analysis, 3- or 4-fold higher amounts of the samples derived from the cultures at 42 °C were loaded on the gel (Figure 4A, see lane 5 and lane 6).

LPS on a duplicate SDS-PAGE gel was transferred to a PVDF membrane. Molecules on the upper portion of the membrane transferred from the 12% SDS-PAGE gel were hybridized with the monoclonal antibodies (mAb) MF15-4 against O-antigen (Figure 4B). On the other hand, molecules on the lower membrane portion transferred from the 15% SDS-PAGE gel were first hybridized with mAb 26-5 against lipid A (Figure 4C) and subsequently re-hybridized with mAb 5C7-4 against the inner core after stripping (Figure 4D). To compare the relative localization of the lipid A and inner core signals, the pseudo-colored blot images were superimposed based on the protein markers. The result showed that lipid A migrated in front of the inner core (Figure 4E). We found that lipid A, core oligosaccharide, and O-antigen were not detected in *lpxH(Ts)* at the restrictive temperature (Figure 4, see lane 4 and lane 6). This result suggests that *lpxH* in *P. aeruginosa* is essential for lipid A biosynthesis, similar to that of *lpxH* in *E. coli* [22]. Additionally, without lipid A or a membrane anchor of LPS, other components such as core oligosaccharide and O-antigen were also missing in *lpxH(Ts)* at the restrictive temperature.

### 3.5. Expression of the Non-Homolog UDP-2,3-Diacylglucosamine Hydrolyses Cc.LpxI and Ct.LpxG Compensate for the Loss of LpxH in P. aeruginosa

**Figure 5:**
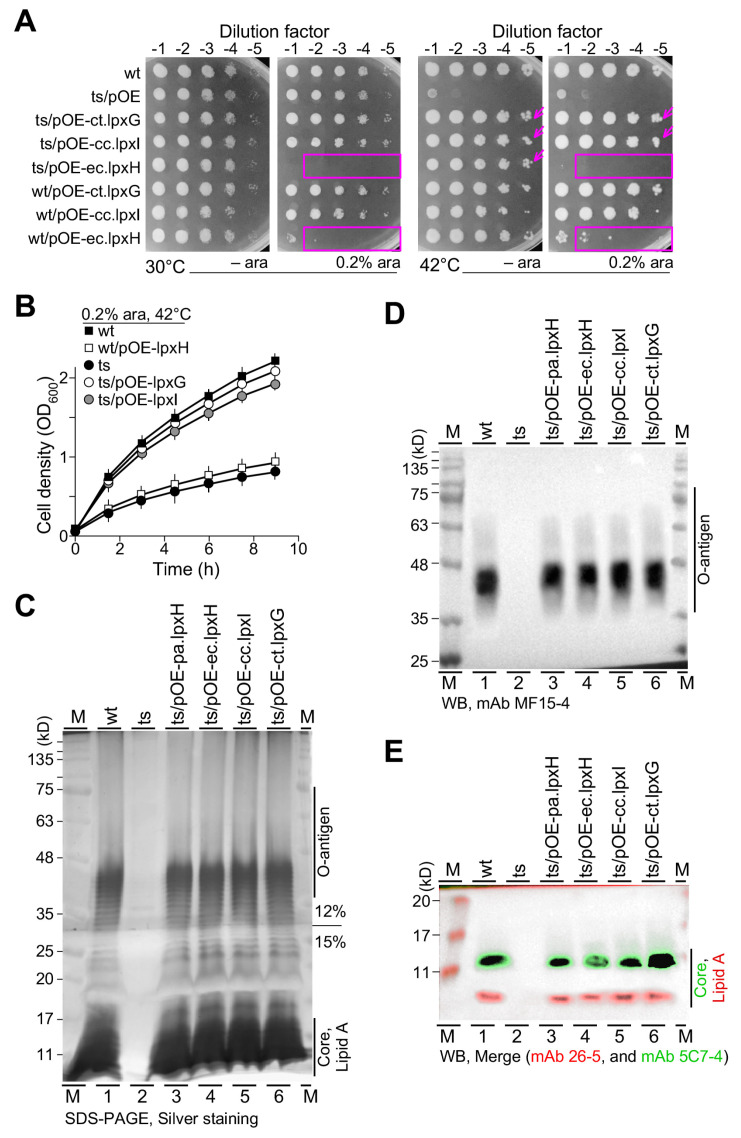
*lpxH(Ts)* is rescued by the leaky and strong expression of *lpxI* or *lpxG* and by the leaky but not strong expression of *lpxH*. (**A**) Spot-plating assay. Leaky expression level of *ec.lpxH*, *cc.lpxI*, and *ct.lpxG* rescues *lpxH(Ts)* at 42 °C (see arrow). Strong expression level of *lpxH*, but not *lpxI* or *lpxG* impedes cell growth (see rectangle). (**B**) Growth curve analysis. X- and Y-axes indicate time (h) and cell density (OD_600_). (**C**) Silver-stained SDS-PAGE gel image. (**D**) Image of Western blot probed with mAb MF15-4 against O-antigens. (**E**) Merged image of Western blot probed with mAb 26-5 against lipid A (red) and re-probed with mAb 5C7-4 against the inner core (green).

The non-homologous UDP-2,3-diacylglucosamine hydrolyses such as Cc.LpxI in *Caulobacter crescentus* and Ct.LpxG in *Chlamydia trachomatis* were found to be able to compensate for the loss of LpxH in *E. coli* [11,12]. To investigate whether these non-homologous UDP-2,3-diacylglucosamine hydrolyses *cc.lpxI* and *ct.lpxG* could also compensate for the loss of *lpxH* in *P. aeruginosa*, we constructed the overexpression plasmids pOE-cc.lpxI and pOE-ct.lpxG, similar to that of pOE-ec.lpxH (see Figure 3). The spot-plating assay indicated that the expression of *cc.lpxI* and *ct.lpxG* with or without arabinose rescued the growth defect of *lpxH(Ts)* at the restrictive temperature (Figure 5A, see arrow). Unlike the homolog *ec.lpxH*, strong overexpression of the non-homologs *cc.lpxI* and *ct.lpxG* was unable to impede cell growth, suggesting a subtle difference between the non-homologous isofunctional enzymes LpxH and LpxI or LpxG. We found that growth curve analysis was consistent with the spot-plating assay (Figure 5B).

SDS-PAGE and Western blotting analysis showed that the expression of homolog *ec.lpxH* and non-homologs *cc.lpxI* and *ct.lpxG* restored the lipid A biosynthesis in *lpxH(Ts)* at the restrictive temperature (Figure 5C–E and Figure S1), indicating that the expression of homolog or non-homolog enzymes UDP-2,3-diacylglucosamine hydrolyses not only rescues the growth defect but also restores the biosynthesis of lipid A in *lpxH(Ts)* at the restrictive temperature.

### 3.6. Lethality of lpxH(Ts) at the Restrictive Temperature Is Suppressed by a Sup Mutant Identified from Spontaneous Mutagenesis

**Figure 6:**
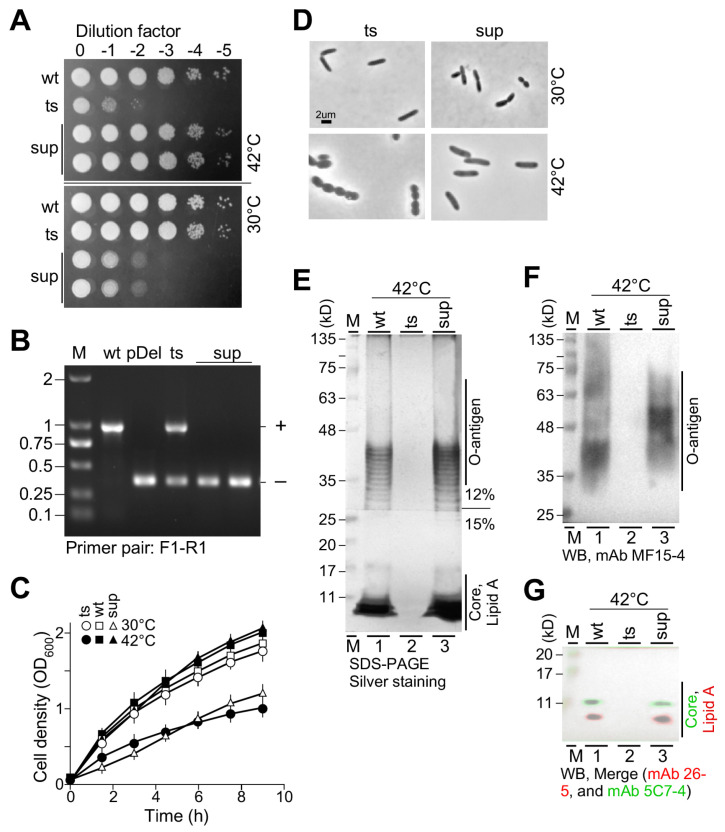
Characterization of a putative suppressor *sup* of *lpxH*. (**A**) Spot-plating assay. (**B**) PCR assay showing the *sup* mutant lacking a wild type allele. (**C**) Growth curve analysis. (**D**) Cell morphology analysis. Oval-shaped morphology was restored to rod-shaped cells in *sup* at 42 °C. At 30 °C, *sup* exhibits oval-shaped morphology. (**E**) Silver-stained SDS-PAGE gel analysis. (**F**) Western blotting image. Molecules derived from the upper portion of the SDS-PAGE gel are probed with mAb MF15-4 against O-antigen. (**G**) Merged image of Western blots. Molecules derived from the lower portion of the SDS-PAGE gel are probed with mAb 26-5 against lipid A (red) and re-probed with mAb 5C7-4 against the inner core (green).

To investigate whether or not *lpxH* mutation could be suppressed, we performed a spontaneous mutagenesis by spreading the *lpxH(Ts)* mutant cells (>1.0 × 10^+09^) on LB plates incubated at the semi-restrictive temperature of 40 °C as we did previously [13,14]. The spot-plating assay confirmed that the *sup* mutant could grow at 42 °C as that of the wild type (Figure 6A). However, *sup* failed to growth at 30 °C, suggesting that mutations in an unknown suppressor gene were temperature sensitive. This could be explained that at the restrictive temperature, the rescue plasmid pTS-lpxH in *lpxH(Ts)* was lost and *sup* was defective, which rescued the lethality of *ΔlpxH*. In contrast, at the permissive temperature, *sup* was effective and failed to suppress the growth defect of *ΔlpxH*. 

To test this possibility, we showed that only the *ΔlpxH* allele but no wild type allele was detected in the *sup* mutant based on the PCR assay (Figure 6B). Growth curve analysis supported the observation in the spot-plating assay. Microscopic analysis showed that *sup* exhibited rod-shaped morphology at 42 °C, which restored the oval-shaped morphology in the *ΔlpxH* mutant (Figure 6D, see bottom row). In contrast, when *sup* cells were shifted from 42 °C to 30 °C, oval-shaped cells started to appear, suggesting that it failed to suppress *ΔlpxH* whose phenotype started to appear (Figure 6D, see upper row).

Subsequently, we investigated whether or not lipid A biosynthesis in *sup* was restored at 42 °C. For this reason, we performed SDS-PAGE and Western blotting analysis. The analysis indicated that the O-antigen, core, and lipid A were all detected in *sup* at 42 °C, indicating that lipid A biosynthesis was restored in the *sup* mutant (Figure 6E–G and Figure S2). However, we were unable to identify the *sup* gene sequence using referenced genome re-sequencing analysis. It was possible that the *sup* locus in the strain we studied was not identical to that in the reference genome of PAO1 [25].

## 4. Discussion

In this study, by using a plasmid-based *ts*-mutant *lpxH(Ts)* in *P. aeruginosa* PAO1, we showed that *lpxH(Ts)* failed to grow at the restrictive temperature with an oval-shaped terminal morphology (see Figure 1 and Figure 2). It has been shown that not all UDP-2,3-diacylglucosamine hydrolyses in Gram-negative bacteria are encoded by *lpxH* [11,12]. Non-homologous *lpxI* in *C. crescentus* and *lpxG* in *C. trachomatis* are found to encode the isofunctional enzyme UDP-2,3-diacylglucosamine hydrolase [11,12].

Though both LpxH and LpxI catalyze UDP-Diacylglucosamine hydrolysis to form lipid X and UMP, the former attacks the α-phosphate and the latter targets the β-phosphate during the catalytic reaction [11]. Like LpxH, LpxG also attacks α-phosphate to hydrolyze UDP-Diacylglucosamine [12]. We show, in this study, the leaky expression level of the araC-P_BAD_ promoter-controlled *lpxH* in *P. aeruginosa* and *E. coli* and their non-homologous isofunctional sequences; *lpxI* in *C. crescentus* and *lpxG* in *C. trachomatis* rescues the lethality of *lpxH(Ts)* at the restrictive temperature (see Figure 5). This result suggests that the release of lipid X from UDP-Diacylglucosamine is sufficient for continuing the Raetz pathway regardless of the α-phosphate or β-phosphate it attacks. 

However, inconsistent with this, under the strong expression level (i.e., induced by 0.2% arabinose supplementation), *lpxH*, but not *lpxI* or *lpxG*, impedes cell growth (see Figure 5). It is known that the strong expression of *lpxG* impedes growth by cell lysis in *C. trachomatis* [12]. These results indicate that the strong expression of *lpxH* and *lpxG* hinders growth and leads to cell lysis in *P. aeruginosa* and *C. trachomatis*, respectively (This study, [12]). We propose that this toxic effect by the strong expression of *lpxH* and *lpxG* requires *lpxH*- and *lpxG*-specific cofactors in *P. aeruginosa* and *C. trachomatis*, respectively. This would explain why the strong expression of *lpxG* shows no deleterious effect in *P. aeruginosa* (see Figure 5).

It has been shown that disruption of the first two genes *lpxA* and *lpxC* in the Raetz pathway is suppressible by a mutation in *fabZ* that is involved in FASII [26]. However, it is unclear whether or not other essential genes in the pathway are suppressible. In this study, we have tested the suppressibility of *lpxH* by suppression analysis using the *ts*-mutant *lpxH(Ts)* (see Figure 6). Though we isolated a putative *sup* colony which survived at 42 °C, we failed to identify the *sup* gene sequence currently.

In conclusion, by using the plasmid-based temperature-sensitive mutant *lpxH(Ts)*, we experimentally confirmed that *lpxH* in *P. aeruginosa* is essential for viability. LpxH is required for lipid A biosynthesis and rod-shaped growth. The strong expression of *lpxH* but not the non-homologous isofunctional UDP-2,3-diacylglucosamine hydrolase impedes growth and causes cell lysis in *P. aeruginosa*. Our study provides insight into the genetic basis of *lpxH* function at various expression levels. We propose that the plasmid-based temperature-sensitive mutant is a useful tool for the genetic analysis of essential genes in *P. aeruginosa*.

## Figures and Tables

**Table 1:** Oligonucleotides, plasmids, and strains used in this study.

**(A) Oligonucleotides**		
**Name**	**Sequence (5′-3′)**	**Usage**
F1	TGATCACGATCATTCCTTGATGC	Assay lpxH alleles in chr and TS-plasmid
R1	TGGACGTGGTCAACAAGATCAAG	Ditto
F2	TTCCTTGCGCTTGATCAGGTAC	Assay lpxH alleles in chr but not TS-plasmid
R2	CCGATGTGCAGGTAACCGTTG	Ditto
**(B) Plasmids**		
**Name**	**Relevant Genotype**	**Reference**
pDEL	pUC-Gm^r^-sacB	[13,14]
pRES or pTS	pUC-Tc^r^-ori^ts^	[13,14]
pOE	pBBRMCS-5-araC-P_BAD_-Gm^r^	[13,14]
pDEL-lpxH	*lpxH* deletion cassette in pDEL	This study
pRES-lpxH	*lpxH* rescue cassette in pTS	This study
pOE-pa.lpxH	araC-P_BAD_-pa.lpxH in pOE	This study
pOE-ec.lpxH	araC-P_BAD_-ec.lpxH in pOE	This study
pOE-cc.lpxI	araC-P_BAD_-cc.lpxI in pOE	This study
pOE-ct.lpxG	araC-P_BAD_-ct.lpxG in pOE	This study
**(C) Strains**		
**Name**	**Relevant Genotype/Usage**	**Reference**
PAO1	Wild type	[13,14]
*ΔlpxH/pTS-lpxH*	*lpxH(Ts)* ts-allele	This study
*ΔlpxH/pTS-lpxH/pOE-lpxH*	*lpxH-OE* in ts	This study
*ΔlpxH/pTS-lpxH/pOE-ec.lpxH*	*ec.lpxH-OE* in ts	This study
*ΔlpxH/pTS-lpxH/pOE-cc.lpxI*	*cc.lpxI-OE* in ts	This study
*ΔlpxH/pTS-lpxH/pOE-ct.lpxG*	*ct.lpxH-OE* in ts	This study
*wt/pOE-lpxH*	*lpxH-OE* in wt	This study
*wt/pOE-ec.lpxH*	*ec.lpxH-OE* in wt	This study
*wt/pOE-cc.lpxI*	*cc.lpxI-OE* in wt	This study
*wt/pOE-ct.lpxG*	*ct.lpxH-OE* in wt	This study
*sup*	*sup ΔlpxH*	This study

## Data Availability

All data supporting the conclusion of this article are included within the manuscript.

## Supplementary Materials

The following supporting information can be downloaded at: https://www.mdpi.com/article/10.3390/genes15060784/s1. Figure S1. Individual blots are used in Figure 5E. Figure S2. Individual blots are used in Figure 6G.
