# Supplementary material for: Genetic Analysis of the Plasmid-Based Temperature-Lethal Mutant pa1792|lpxH(Ts) in Pseudomonas aeruginosa
Source: Genes (Basel). 2024 Jun 14;15(6):784. doi: 10.3390/genes15060784 (PMC11202943; doi:10.3390/genes15060784)

Supplementary Materials

Figure S1. Individual blots are used in Figure 5E.

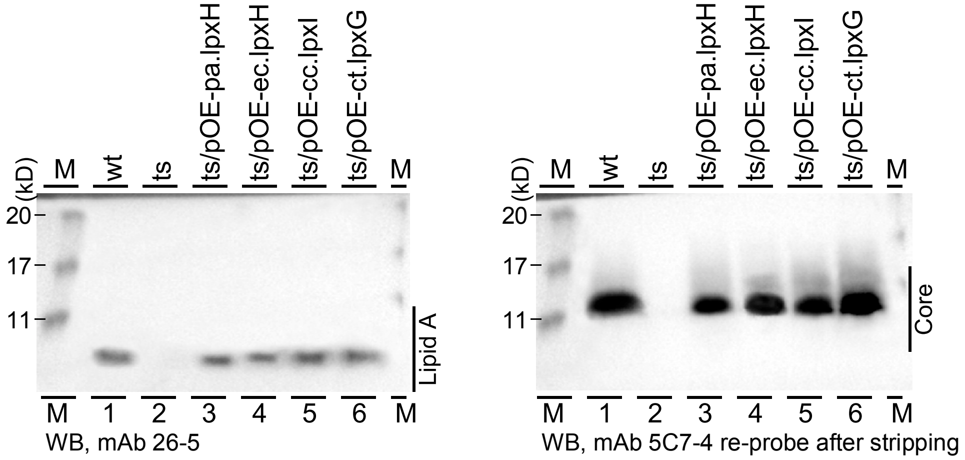

Figure S2. Individual blots are used in Figure 6G.

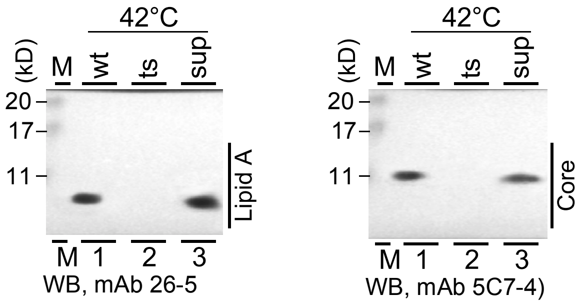

Supplement: Supplementary file 1 [file genes-15-00784-s001.zip › genes-3048160-supplementary.pdf]
